# Frequency and predictors of miliary tuberculosis in patients with miliary pulmonary nodules in South Korea: A retrospective cohort study

**DOI:** 10.1186/1471-2334-8-160

**Published:** 2008-11-26

**Authors:** Sang-Man Jin, Hyun Ju Lee, Eun-Ah Park, Ho Yun Lee, Sang-Min Lee, Seok-Chul Yang, Chul-Gyu Yoo, Young Whan Kim, Sung Koo Han, Young-Soo Shim, Jae-Joon Yim

**Affiliations:** 1 Division of Pulmonary and Critical Care Medicine, Department of Internal Medicine and Lung Institute, Seoul National University College of Medicine, 103 Daehangno Jongno-gu, Seoul 110-744, Republic of Korea; 2 Department of Radiology and Institute of Radiation Medicine, Seoul National University College of Medicine, 103 Daehangno Jongno-gu, Seoul 110-744, Republic of Korea

## Abstract

**Background:**

Miliary pulmonary nodules are commonly caused by various infections and cancers. We sought to identify the relative frequencies of various aetiologies and the clinical and radiographic predictors of miliary tuberculosis (TB) in patients with miliary pulmonary nodules.

**Methods:**

We performed a retrospective cohort study of patients who presented with micronodules occupying more than two-thirds of the lung volume, based on computed tomography (CT) of the chest, between November 2001 and April 2007, in a tertiary referral hospital in South Korea.

**Results:**

We analyzed 76 patients with miliary pulmonary nodules. Their median age was 52 years and 38 (50%) were males; 18 patients (24%) had a previous or current malignancy and five (7%) had a history of TB. The most common diagnoses of miliary nodules were miliary TB (41 patients, 54%) and miliary metastasis of malignancies (20 patients, 26%). Multivariate analysis revealed that age ≤30 years, HIV infection, corticosteroid use, bronchogenic spread of lesions, and ground-glass opacities occupying >25% of total lung volume increased the probability of miliary TB. However, a history of malignancy decreased the probability of miliary TB.

**Conclusion:**

Miliary TB accounted for approximately half of all causes of miliary pulmonary nodules. Young age, an immune-compromised state, and several clinical and radiographic characteristics increased the probability of miliary TB.

## Background

Miliary pulmonary nodules are commonly caused by various infections and cancers. In fact, a heterogeneous groups of conditions comprising more than 80 entities may result in miliary nodules [1]. Among them, miliary tuberculosis (TB) is one of the most frequent aetiologies in areas with a high prevalence of TB [2]. However, approximately two-thirds of all miliary TB cases are acid-fast bacilli (AFB) smear-negative, and even transbronchial biopsy frequently fails to reveal AFB or caseating granulomas [3-5]. In such cases, the remaining options for the rapid diagnosis of miliary TB include invasive procedures such as open lung biopsy or empirical anti-TB treatment, depending on the probability of miliary TB based on clinical and radiographic findings. Nonetheless, no recent report has examined the role of clinical characteristics in the differential diagnosis of miliary pulmonary nodules. Moreover, the clinical role of radiographic findings in differentiating between miliary TB and other aetiologies of miliary pulmonary nodules has not been established, although differential diagnosis based on the distribution of miliary nodules in high-resolution computed tomography has been studied [2,6-8].

We examined the relative frequencies of various aetiologies of miliary pulmonary nodules and sought to identify clinical and radiographic predictors of miliary TB in a tertiary referral hospital in South Korea, where the incidence of active TB was reported to be about 96/100,000 persons in 2005 [9].

## Methods

### Study subjects

We included all adult patients (≥18 years of age) who presented with miliary nodules, based on computed tomography (CT) of the chest, between November 2001 and April 2007. Patients were selected from Seoul National University Hospital, a tertiary referral hospital in South Korea. Miliary nodules were defined as micronodules (<7 mm in diameter [10]), regardless of solid or non-solid nature, occupying more than two-thirds of the lung volume on a conventional chest CT or high-resolution chest CT. We excluded patients whose physician had not fully evaluated the cause of the miliary nodules, patients who defaulted before obtaining a final diagnosis, and patients with concurrent discrete macronodules (>7 mm in diameter). However, patients with concurrent consolidations were included.

The study protocol was approved by the institutional review board of Seoul National University Hospital.

### Study design

We performed a retrospective cohort study to examine the relative frequencies of aetiologies of miliary pulmonary nodules and to assess clinical and radiographic predictors of miliary TB. Enrolled patients were classified into two groups, miliary TB and miliary nodules due to other causes, according to the final diagnosis of their miliary pulmonary nodules. To reveal predictors of miliary TB, clinical and radiographic characteristics were analyzed between the groups.

### Review of clinical findings and laboratory tests

We retrospectively reviewed the demographic and clinical characteristics, including age, gender, body mass index (BMI), and symptoms, of the selected patients. In addition, we checked for the presence of previous TB, malignancy, diabetes mellitus, immunosuppressive drug therapy, solid organ transplantation, connective tissue disease, chronic kidney disease, chronic liver disease, HIV infection, and alcoholism. We also reviewed the results of laboratory tests, including complete blood cell counts, blood chemistry, and C-reactive protein levels. The results of histopathological examinations and microbiological studies, including acid-fast staining, mycobacterial cultures, and fungal cultures, were also reviewed.

### Radiographic evaluations

Chest CT scans performed at the time of diagnosis were reviewed by two board-certified radiologists, by consensus. They were blinded to patient clinical data and had not participated in the case selection.

### Characteristics and distribution of miliary nodules

Miliary nodules were assessed for size, margin, profusion, and dominant distribution. Considering previous reports that miliary nodules from cancer usually consist of micronodules of variable size or relatively large micronodules (4–7 mm in diameter) [1,11], we described the size of the miliary nodules as 'homogeneous' when the sizes of all micronodules were confined to 0–4 mm. The profusion of nodules was estimated by counting all nodules in two contiguous squares of 4 cm^2 ^on three levels: just above the aortic arch, the right upper lobar bronchus, and the lower portion of the left atrium [7]. The dominant distribution of miliary nodules within secondary pulmonary lobules was described as centrilobular, perilymphatic, or random [1,2,6,11].

### Definition of bronchogenic spread and pre-existing tuberculosis (TB) sequelae

Poorly defined centrilobular nodules, branching linear lesions, tree-in-bud appearance, and bronchial wall thickening were classified as indicating the bronchogenic spread of lesions [7]. Patients with fibrotic bands, small calcified nodules, or bronchiectasis observable in the upper lobes were regarded as having pre-existing TB sequelae [12,13]. Mediastinal lymph nodes >1 cm in short-axis diameter were considered positive signs of lymphadenopathy. Additionally, we assessed the extent of accompanying ground-glass opacities, consolidations, and pleural effusions.

### Diagnostic criteria

Miliary TB was confirmed if any of the following was met: (1) positive staining for AFB, the growth of *Mycobacterium tuberculosis*, or positive PCR for *M. tuberculosis *DNA in sputum, bronchial lavage fluid, pleural fluid, or tissue; (2) the presence of caseating granulomas in lung tissue and clinical and radiographic improvement with anti-TB medications; or (3) definite clinical and radiographic improvement with empirical anti-TB medications. Miliary metastasis of malignancies was identified when any of the following was met: (1) cytological/histological examination of miliary nodules showing malignant cells; or (2) concurrent changes in the size and number of miliary nodules relative to the size of measurable primary malignant lesions. For example, concurrent decreases in the sizes of both primary lesions and miliary nodules in a patient receiving anti-cancer chemotherapy would be such a case. Pneumoconiosis was diagnosed based on occupational history and compatible clinical and radiographic findings. Disseminated fungal diseases were defined using bacteriological or pathological analyses of miliary nodules. Hypersensitivity pneumonitis was diagnosed by environmental or occupational history and improvement with environmental control or corticosteroid treatment.

### Statistical analyses

To identify factors for differentiating between TB-associated miliary nodules and miliary nodules due to other causes, we performed univariate analyses using the χ^2 ^test or Fisher's exact test for categorical variables, and using *t *tests for continuous variables. To determine predictors of miliary TB, we performed binary logistic regression analysis with significant (*P *< 0.05) variables from the univariate analysis. Co-linearity was eliminated by stepwise selection (entry, *p *= 0.05; removal, *p *= 0.1). Because some predictors were likely to be found only within the miliary TB group, we used the penalized likelihood ratio test to determine significance. For the same reason, odds ratios and 95% confidence intervals were estimated using the penalized maximum likelihood and profile penalized likelihood confidence intervals, respectively. Statistical significance was determined at *P *< 0.05. All analyses were performed using SPSS (version 12.0, SPSS Inc., Chicago, IL, USA) or SAS (version 8.2, SAS Institute Inc., Cary, NC, USA).

## Results

### Demographic and clinical characteristics of patients with miliary pulmonary nodules

A total of 182 patients diagnosed with and treated for miliary pulmonary nodules during the study period were initially screened for inclusion in this study. Of these, 102 patients were excluded because of concurrent discrete macronodules (>7 mm in diameter), mass lesions suggestive of primary lung cancer, or cavitations suggestive of pulmonary TB. Indeed, the diagnoses of the 102 patients excluded from further analysis were metastatic cancer (65 patients), miliary TB (29 patients), and pneumoconiosis (8 patients). An additional four patients were excluded because they defaulted before obtaining a final diagnosis. Finally, data for 76 patients were included and analyzed. Their median age was 52 years and 38 (50%) of them were males; 18 patients (24%) had a previous or current malignancy and five (7%) had a history of TB (Table 1).

**Table 1:** Demographic and clinical characteristics of 76 patients with miliary nodules.

Age (yr), median (range)	52 (21–86)
Male	38 (50%)
Body mass index (kg/m^2^), mean ± SD	21.8 ± 3.0*
Smoking history	
Current smoker	12/74 (16%)
Ex-smoker	3/74 (4%)
Median pack-years (range)	30 (3–50)
Body temperature > 37.8°C	21/61 (34%)
Leukocytosis (>10,000/mm3)	11/72 (15%)
C-reactive protein (mg/dL), mean ± SD	4.9 ± 5.6^†^
Previous or current malignancy	18 (24%)
Solid organ cancer	12
Hematologic malignancy	6
History of previous TB	5 (7%)
Comorbidities	
Diabetes mellitus	2 (3%)
HIV infection	7 (9%)
Corticosteroid use^‡^	9 (12%)
Connective tissue disease	6 (8%)
Solid organ transplantation	2 (3%)
Chronic kidney disease^§^	2 (3%)

* Result of 61 subjects

^† ^Result of 55 subjects

‡ Prednisolone equivalent ≥5 mg/d for >3 wk.

^§^Glomerular filtration rate <60 mL/min/1.73 m^2 ^for 3 or more months.

### Aetiologies of miliary pulmonary nodules

Among the 76 patients, miliary TB accounted for 54% of the miliary pulmonary nodules (41 patients). Of these, 31 patients (76%) were diagnosed by positive staining for AFB, the growth of *Mycobacterium tuberculosis*, or positive PCR for *M. tuberculosis *DNA in sputum, bronchial lavage fluid, pleural fluid, cerebrospinal fluid, or tissue. In five patients (12%), diagnoses were made by the presence of caseating granulomas in lung tissue and clinical and radiographic improvement with anti-TB medications. The other five patients (12%) were diagnosed based on definite clinical and radiographic improvement with empirical anti-TB medications. Miliary metastasis of malignancies accounted for 26% of the miliary pulmonary nodules (20 patients). Of these, direct histological confirmation of miliary pulmonary nodules via lung biopsy was achieved in one patient. Three patients were diagnosed with disseminated candidiasis, all with an underlying hematologic malignancy (Table 2).

**Table 2:** Etiologies of 76 patients with miliary nodules.

Tuberculosis	41 (54%)
Disseminated candidiasis	3 (4%)
Malignancy	20 (26%)
Lung cancer*	7
Breast cancer	5
Thyroid cancer	2
Others^†^	6
Pneumocconiosis	6 (8%)
Sarcoidosis	4 (5%)
Hypersensitivity pneumonitis	2 (3%)

*Six of them had adenocarcinoma of non-bronchioloalveolar carcinoma histology and the other had bronchioloalveolar carcinoma.

^†^Advanced gastric cancer (1 patient), prostate cancer (1 patient), non-Hodgkin's lymphoma (1 patient), pancreas cancer (1 patient), parotid gland adenoid cystic carcinoma (1 patient), thymic carcinoma (1 patient)

### Clinical and radiological findings associated with miliary TB

With univariate analysis, we found that age ≤30 years, HIV infection, corticosteroid use, connective tissue disease, low BMI, body temperature >37.7°C, homogeneous nodule size, high profusion, bronchogenic spread of lesions, and ground-glass opacities occupying >25% of total lung volume were associated with miliary TB. However, a history of solid organ malignancy decreased the probability of miliary TB (Tables 3, 4). Subsequent multivariate analysis supported the association between miliary TB and age ≤30 years, HIV infection, corticosteroid use, bronchogenic spread of lesions, and ground-glass opacities occupying >25% of total lung volume, as well as the association between miliary nodules of non-miliary TB and a history of malignancy (Table 5).

**Table 3:** Univariate analysis of clinical characteristics between patients with miliary tuberculosis and other diseases

	TB (n = 41)	Other diseases (n = 35)	*P *value
Age ≤30	10 (24%)	1 (3%)	.008
Male	22 (54%)	16 (46%)	.490
Body mass index (kg/m^2^), mean	20.6 ± 3.2 (n = 32)	23.1 ± 2.2 (n = 29)	.001
Ever-smoking	5/40 (13%)	10/34 (29%)	.197
Body temperature > 37.7°C	19/39 (49%)	2/22 (9%)	.002
Leukocytosis	6/40 (15%)	5/32 (16%)	1.000
C-reactive protein (mg/dL)	5.9 ± 4.8 (n = 35)	3.1 ± 6.5 (n = 20)	.070
Presence of classic risk factor for miliary TB*			
History of malignancy	2 (5%)	16 (46%)	<.001
Solid organ cancer	0	12 (34%)	<.001
Hematologic malignancy	2 (5%)	4 (11%)	.405
History of previous TB	4 (10%)	1 (3%)	.366
Diabetes mellitus	2 (5%)	0	.496
HIV infection	7 (17%)	0	.013
Corticosteroid use^†^	9 (22%)	0	.003
Connective tissue disease	6 (15%)	0	.028
Solid organ transplantation	2 (5%)	0	.496
Chronic kidney disease^‡^	2 (5%)	0	.496

*There could be more than one risk factor.

^†^Prednisolone equivalent ≥5 mg/d for >3 wk.

^‡^Glomerular filtration rate <60 mL/min/1.73 m^2 ^for 3 or more months.

**Table 4:** Univariate analysis of radiographic characteristics between patients with miliary tuberculosis and other diseases

	TB (n = 41)	Other diseases (n = 35)	*P *value
Homogeneous nodule size*	30 (73%)	17 (49%)	.028
Dominant distribution			
Random	37 (90%)	30 (86%)	.794
Centrilobular	2 (5%)	2 (6%)^†^	
Perilymphatic	2 (5%)	3 (9%)^‡^	
Profusion (N/cm^2^)			
Upper	5.2 ± 2.7	3.2 ± 2.6	.002
Middle	5.2 ± 2.6	3.1 ± 2.5	.001
Lower	4.6 ± 2.5	2.5 ± 2.5	.001
Overall	5.0 ± 2.4	2.9 ± 2.3	<.001
Sharp margin	30 (75%)	20 (57%)	.102
Bronchogenic spread of lesion	23 (56%)	5 (14%)	<.001
Ground-glass opacity extent			
None	20 (49%)	24 (69%)	.082
0–25%	11 (27%)	10 (29%)	.866
25–100%	10 (24%)	1 (3%)	.008
Preexisting TB lesion	5 (12%)	5 (14%)	1.000
Pleural effusion	15 (37%)	10 (29%)	.459
Mediastinal lymphadenopathy	16 (39%)	20 (57%)	.115
Consolidation	17 (42%)	16 (46%)	.709

*Size of all micronodules confined to 0–4 mm

^†^Peumocconiosis (n = 1), hypersensitivity pneumonia (n = 1)

^‡^Sarcoidosis (n = 2), pneumoconiosis (n = 1)

**Table 5:** Multivariate analysis to identify the clinical and radiographic predictors of miliary TB

	Adjusted OR* (95% CI^†^)	*P *value^‡^
Bronchogenic spread of lesion	9.1 (2.1–50.8)	.002
Ground-glass opacity extent >25%	13.3 (2.3–146.1)	.003
Age ≤30	21.2 (2.7–373.2)	.003
History of malignancy	0.1 (0.0–0.7)	.013
Corticosteroid use	20.0 (1.6–2893.8)	.017
HIV infection	19.9 (1.5–2885.3)	.020

*Penalized maximum likelihood

^†^Profile penalized likelihood confidence interval

^‡^Penalized likelihood ratio test

## Discussion

Although the prevalence of active pulmonary TB in South Korea decreased from 5.1% in 1965 to 0.33% in 2005 [14], this study showed that miliary TB still accounted for approximately half of all cases of miliary pulmonary nodules in the patients examined. The proportion of miliary TB among miliary pulmonary nodules in the present study was higher than that in a previous study by Lee et al. (22.5%) in a South Korean population [2]. This discrepancy is probably the result of differences in study design. In the previous study, patients without biopsy-proven diagnoses were not included [2], which might have excluded a considerable portion of miliary TB patients who may have been identified by bacteriological studies.

Our study indicated that age ≤30 years, HIV infection, and corticosteroid use were strong independent predictors of miliary TB (Table 5). Although miliary TB is relatively rare among young adults without HIV infection in developed nations [15], about one-quarter of the non-HIV-infected patients with miliary TB in this study were ≤30 years of age. This difference may be attributable to the relatively high incidence of TB in South Korea, where the annual incidence of active TB has been reported to be about 96/100,000 persons per year [9].

Other risk factors for miliary TB, including diabetes mellitus [3-5], a history of prior TB [4,5], chronic kidney disease [3-5], and hematologic malignancies [16], failed to show an association in our study population (Table 3). These differences may also be attributable to study design. All of the subjects in the present study had miliary pulmonary nodules, and clinical characteristics were subsequently compared between miliary TB patients and patients with other miliary pulmonary nodules. However, previous studies compared clinical characteristics between miliary TB patients and healthy controls *without *TB [3-5]. Certain clinical features are more common in miliary TB patients than in the general population, but these factors may have less significance in the clinical setting of miliary pulmonary nodules of various aetiologies.

A history of hematologic or solid malignancy (OR, 0.1; 95% CI, 0.0–0.7) was a strong negative predictor of miliary TB in our study (Table 5). Although hematologic and solid organ malignancy is a known risk factor for the reactivation of TB [16-22], the presence of a history of malignancy decreased the probability of miliary TB in our study. The miliary nodules found in 13 of 18 patients (72%) with underlying malignancies represented metastases of those underlying malignancies.

Among radiographic findings, this study revealed that the bronchogenic spread of lesions and ground-glass opacities occupying >25% of total lung volume were associated with miliary TB. This confirmed a previous observation that these parameters are frequently found in miliary TB [7,23]. However, a random distribution of miliary nodules on high-resolution computed tomography scans, which is a well-known characteristic of miliary TB [1,2,6-8,23], was not identified as a predictor in this study. This may reflect the fact that all malignant miliary nodules showed a random distribution in our study. Although a random distribution can be helpful in differentiating between miliary TB and diffuse panbronchiolitis [2,11] or sarcoidosis [1,2,6,7,11], its usefulness in diagnosis may be limited in clinical situations where the majority of miliary nodules are caused by miliary TB or malignant metastasis.

To correctly appreciate these results, the limitations of this study should be considered. First, this study was performed in South Korea, where the annual incidence of active TB has been reported to be about 96/100,000 persons per year [9]. Any generalization of the results to patients in areas with higher or lower TB rates should be made with caution. Second, we did not include patients who had miliary nodules but no CT scan, and we excluded patients with discrete lung masses. Thus, the results should be applied cautiously and should not be applied to cases with miliary nodules of clinically apparent causes. For example, a case of miliary nodules with a concurrent spiculated mass highly suggestive of lung cancer would not be applicable to the results of this study. Third, the confidence intervals of the odds ratios for some variables were wide, as a result of the small numbers of patients in some categories. Future studies prospectively recruiting a larger number of patients should be performed to confirm the results of this study.

## Conclusion

In conclusion, although the prevalence of TB has markedly decreased in South Korea, miliary TB still accounts for about half of all causes of miliary pulmonary nodules. To avoid delayed anti-TB treatment, miliary TB should be seriously considered in patients with the factors identified in this study: ≤30 years of age, HIV infection, corticosteroid use, absence of a history of malignancy, bronchogenic spread of lesions, or ground-glass opacities occupying >25% of total lung volume in chest CT scans.

## Competing interests

The authors declare that they have no competing interests.

## Authors' contributions

SMJ collected and analysed the data, drafted and edited the manuscript. HJL designed and advised the radiographic analysis. EAP and HYL reviewed the computed tomography of enrolled patients. SML, SCY, CGY, YWK, SKH, YSS participated in design and contributed to editing the manuscript. JJY conceived of the study, and participated in its design and coordination and helped to draft the manuscript. All authors read and approved the final manuscript.

## Pre-publication history

The pre-publication history for this paper can be accessed here:


